# Changes in human footprint drive changes in species extinction risk

**DOI:** 10.1038/s41467-018-07049-5

**Published:** 2018-11-05

**Authors:** Moreno Di Marco, Oscar Venter, Hugh P. Possingham, James E. M. Watson

**Affiliations:** 1 Centre for Biodiversity and Conservation Science, The University of Queensland, 4072 Brisbane, QLD Australia; 2 CSIRO Land & Water, EcoSciences Precinct, 41 Boggo Road, Dutton Park, QLD 4102 Australia; 3 Natural Resource and Environmental Studies Institute, University of Northern British Columbia, 3333 University Way, Prince George, V2N 4Z9 Canada; 4 The Nature Conservancy, 4245 North Fairfax Drive, Suite 100, Arlington, VA 22203-1606 USA; 5 Global Conservation Program, Wildlife Conservation Society, 2300 Southern Boulevard, Bronx, NK 10460 USA

## Abstract

Predicting how species respond to human pressure is essential to anticipate their decline and identify appropriate conservation strategies. Both human pressure and extinction risk change over time, but their inter-relationship is rarely considered in extinction risk modelling. Here we measure the relationship between the change in terrestrial human footprint (HFP)—representing cumulative human pressure on the environment—and the change in extinction risk of the world’s terrestrial mammals. We find the values of HFP across space, and its change over time, are significantly correlated to trends in species extinction risk, with higher predictive importance than environmental or life-history variables. The anthropogenic conversion of areas with low pressure values (HFP < 3 out of 50) is the most significant predictor of change in extinction risk, but there are biogeographical variations. Our framework, calibrated on past extinction risk trends, can be used to predict the impact of increasing human pressure on biodiversity.

## Abstract (web summary)

Species extinction risk is difficult to measure and often lags behind the pace of increasing threats. Here, the authors demonstrate how monitoring changes in cumulative human pressures could be used to rapidly assess potential change in species’ conservation status.

## Introduction

Species are disappearing at rates that are 1000 times faster than those registered in the fossil record^1^, and accurate predictions of extinction risk are necessary to anticipate declines under past, current, and projected levels of human pressure. Understanding the relationship between changes in human pressures and the decline of individual species is necessary for identifying those species at highest risk, and for prioritising the actions and policies required to combat their decline^2,3^. Comparative extinction risk modelling, which builds on the relationship between species threat status, their life histories, and the pressure mapped within their ranges, is increasingly used to predict the risk of extinction^4–8^. This approach allows inferring the extinction risk of a large number of species based on readily available data, and predictions can be updated more often than expert-based assessments, given the substantially lower resources requirement^9,10^. However, a major limitation in these analyses is the absence of a link to spatial and temporal changes in human pressure and how these lead to change in the risk of species declines^8^. This is further complicated by two types of change in human pressure, the change in extent of pressures (e.g. road building in a new area), and the intensification of existing pressures (e.g. increase in deforestation rates). The missing linkage between pressure and extinction risk means comparative extinction risk analysis has struggled to inform policy and management^11^.

As a species’ conservation status is sensitive to changes in human pressure^12,13^, more dynamic extinction risk modelling has the potential to elucidate links between trends in pressures and trends in extinction risk. The recent publication of a temporally inter-comparable map of human footprint^14^ (HFP) presents an important advance in the global representation of changing human pressure on the terrestrial environment. The map, which incorporates eight pressure layers standardised into a cumulative index (see Methods for details), is calculated at two time points and provides an opportunity to investigate the relationship between changes in human pressure and changes in the status of biodiversity. HFP provides a spatially explicit index of cumulative human pressure ranging from 0 to 50, where a value of zero corresponds to ‘wilderness areas’ free from any significant human influence^15^, a value of four corresponds to low pressure levels (e.g. pasture lands), and values above 20 typically represents very high pressure levels (e.g. densely populated semi-urban and urban areas)^14^. Yet, the HFP is not necessarily a direct measure of threat to species, and it would be inappropriate to assume that all species respond to human activities in the same way. Consequently, the relationship between HFP and species extinction risk requires testing, in the context of environmental and life-history characteristics of each species.

**Fig. 1:**
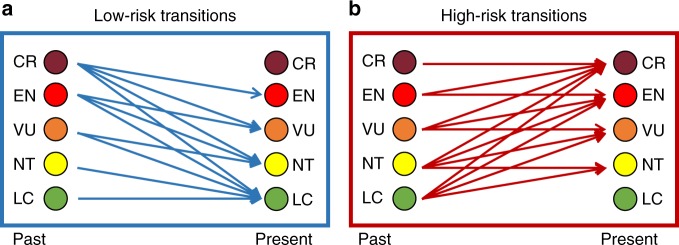
Classification of species extinction risk transitions, based on past and present IUCN Red List categories*. Low-risk transitions include those species that were of least concern throughout the study period, together with species that moved from any higher category of threat to a lower one. High-risk transitions include all species that were originally threatened or near threatened and retained their category throughout the study period, together with species that moved from any lower category of threat to a higher category. *Acronyms for the Red List categories: Least Concern (LC); Near Threatened (NT); Vulnerable (VU); Endangered (EN); Critically Endangered (CR)

Here we compare a 16-year trend in HFP (1993–2009) with a 12-year trend in the extinction risk of 4421 terrestrial mammal species (1996–2008). Our goal is to test the existence of a direct relationship between changing human pressure, as represented by the HFP, and changing risk status of species over a comparable time frame. This allows for the dynamic, as opposed to static, modelling of species extinction risk^8^, and takes advantage of a single, cumulative, representation of how human pressure has changed over time^16^. We focus on terrestrial mammals as they have had their extinction risk measured over a similar period as HFP^13^, and they have served as a focal group in several previous extinction risk analyses^17^. We classified species into two groups, following earlier work^8^: ‘low-risk’ transitions and ‘high-risk’ transitions (Fig. 1). The low-risk group included species that retained a category of least concern and species that moved from any higher category of threat to a lower category during the study period. The high-risk group included all species that retained a category of threatened or near threatened, together with species that moved from any lower category of threat to a higher category. We also test a more conservative classification of risk change, where species are considered either ‘uplisted’, if they moved from any Red List category to a higher category during the study period, or ‘not uplisted’. We measured the proportion of each species’ range overlap with high HFP values, and how this overlap has changed through time, testing all possible definitions of what constitutes ‘high HFP’. We used these values, and other known human pressure, environmental, and life-history predictors of risk (Table 1), to provide estimate of the extinction risk transitions of species as a function of change in human pressure within their distributions.

**Table 1:** Description of the variables used to predict extinction risk transitions in the random forest model

Class	Variable	Description and source	Source
Pressure	High HFP extent	Proportion of species range overlapping with high human footprint values in 2009.	^14^
Pressure	High HFP change	Difference in the proportional overlap between species range and high HFP during 1993–2009.	^14^
Pressure	Human population density	Density of human population in year 2000.	^46^
Pressure	Human population growth	Human population growth, proportional increase in human population between 1990 and 2000.	^47^
Pressure	Travel time to cities	Accessibility from major cities, measured as travel time.	^48^
Life history	Taxonomic order	Species taxonomic orders.	^35^
Life history	Gestation length	Gestation length, a proxy of species reproductive output.	^49, 50^
Life history	Weaning age	Weaning age, a proxy of species reproductive timing.	as above
Life history	Body mass	A generic proxy of species life history and energetic requirements	^49– 51^
Life history	Diet	Dietary category: carnivore ( > 90% animal matter ingested), omnivore (10–90%), herbivore ( < 10%).	^52^
Life history	Habitat class	Species preferences of macro-habitat categories: aquatic, artificial, caves, desert, forest, grassland, rocky areas, savanna, shrubland, generalists (two or more of the previous categories).	^35, 53^
Environment	NDVI	Normalized difference vegetation index, proxy of primary productivity, registered from year 2013.	^54^
Environment	Tree cover	Percentage tree cover values registered in year 2000.	^55^
Environment	Habitat prevalence	Proportion of suitable habitat within species range.	^53^

Variables are aggregated into three main classes (human pressure, life-history, environmental characteristics)

Our results show the importance of HFP as a predictor of extinction risk transition in terrestrial mammals, and suggest the conversion of natural and semi-natural areas (those with low HFP values) has the strongest association with high-risk transitions in species conservation status. We also identified biogeographical differences in the best HFP threshold to determine areas of ‘high pressure’, which can be used for regional monitoring of extinction risk change.

## Results

### Global change in human pressure and species extinction risk

**Fig. 2:**
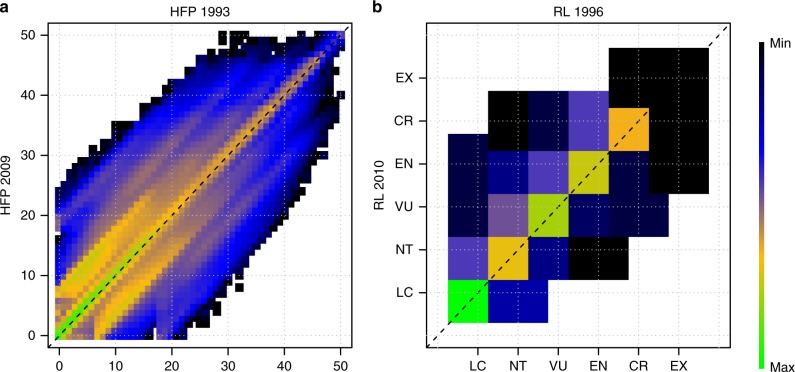
Recent changes in terrestrial human footprint and species extinction risk. **a** Shows a transition matrix in which any position represents the initial (*x* axis) and final (*y* axis) human footprint value (from 0 to 50) of global 1 km terrestrial grid cells; the colour scheme represents the number of individual cells in each particular transition state. **b** Shows a transition matrix in which any position represents the initial (*x* axis) and final (*y* axis) extinction risk category (from Least Concern to Extinct) of terrestrial mammal species; the colour scheme represent the number of individual species in each particular transition state

Much of Earth’s terrestrial surface (30.8%) has undergone an increase in human pressure, as indicated by HFP values that have increased since 1993 (Fig. 2a; Supplementary Fig. 1a). Two thirds of those areas already had relatively high HFP values^18^ in 1993 ( ≥ 4) which became even higher by 2009. At the other end of the spectrum, most of the areas that did not face an increase in human pressure (41.5% of the total terrestrial surface) are characterised by a relatively low HFP value ( < 4). Half of these low-HFP areas have been identified as the last remaining terrestrial ‘wilderness’, which is free of any significant human disturbance (HFP = 0).

When looking at the transitions in species extinction risk, we found that 69% of species faced a low-risk transition, while 31% faced a high-risk transition (Fig. 2b; Supplementary Fig. 1b). This is largely due to 1,229 (27.8%) threatened and near-threatened species retaining their Red List category, and in minor part to 159 uplisted species (3.6%) that moved towards higher Red List categories. Only 22 (0.5%) species moved towards lower Red List categories during the study period.

### Measuring human pressure within species ranges

**Fig. 3:**
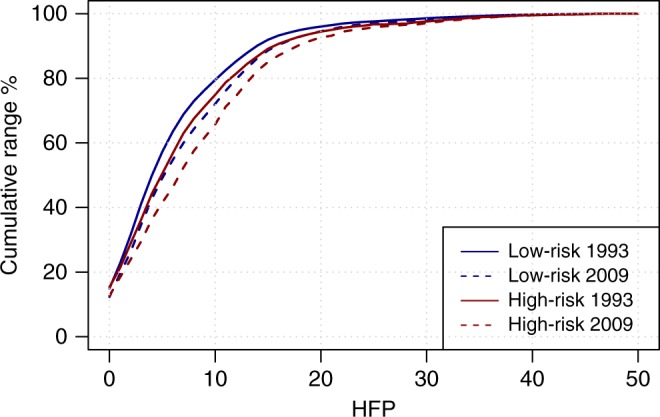
Cumulative extent of human footprint values within mammal species ranges. The lines represent the cumulative percentage of species range that overlaps with increasing values of human footprint, averaged among all species. Different lines refer to species in the low-risk or high-risk categories, for the period 1993 or 2009, as reported in legend

The cumulative distribution of HFP values within species geographic ranges followed similar patterns across the two species groups (high-risk and low-risk) and across years (Fig. 3). However, high-risk species had on average a larger proportion of their range overlapping with high HFP values, compared to low-risk species. The level of overlap with those areas classified as wilderness was comparable between high-risk and low-risk species, while the biggest differences among the two groups was observed for HFP values in the range 3–15, which correspond to moderate or high levels of human pressure^16^. This was reflected in significantly higher mean HFP values within the range of high-risk species compared to low-risk species (*p*-value = 2*10^−12^ in 1993 and 2* 10^−16^ in 2009; Wilcoxon signed rank test, one-sided; Supplementary Fig. 2).

**Fig. 4:**
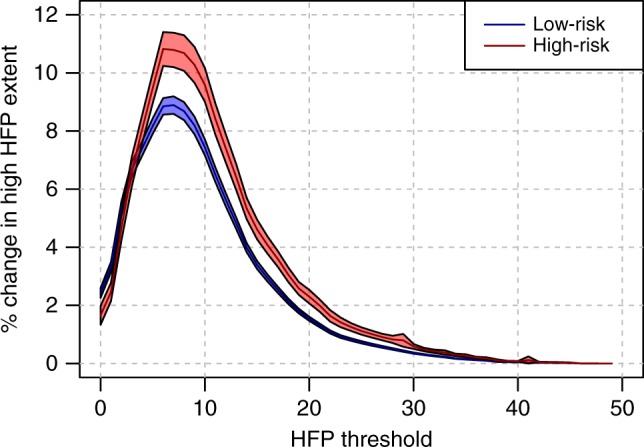
Changes in the overlap between species ranges and high human footprint values over time. The lines report the average change in the overlap between species ranges and human footprint values bigger than any given threshold. Different lines refer to species in the low-risk or high-risk categories, as reported in legend. The shaded areas around the lines represent the 95% standard credible interval measured across a total of 4421 species

High-risk species typically faced a larger change in the extent of high HFP values compared to low-risk species, with the exceptions of very low HFP thresholds (Fig. 4). When looking at HFP thresholds between 0 and 2, low-risk species had a larger proportion of their range moving from below- to above-threshold values compared to high-risk species. This might be related to many threatened and declining species retaining little natural areas within their range at the beginning of the study period, with consequent little chances of observing an increasing HFP in natural areas during the study period. For HFP in the range 3–49, high-risk species consistently showed higher proportions of their range moving to above-threshold values, with a difference that was significant for thresholds in the range 6–44 (*p*-values = 2*10^−6^–2*10^−2^; Wilcoxon signed rank test, one-sided). Overall, the largest effect size for the difference in extent of high HFP values between low-risk *vs* high-risk species was observed for a HFP threshold of 3 (Cohen’s d = 0.43) and decreased afterwards, while the effect size for change in the extent of high HFP values increased up to a threshold of 6 and then stabilized (with Cohen’s d values in the range 0.20–0.22; Supplementary Fig. 3 and 4).

When looking at the difference in extent of high HFP values for uplisted vs. not uplisted species, we found even larger differences than those described for low-risk vs. high-risk species (Supplementary Fig. 5), with substantially higher values for uplisted species when looking at HFP thresholds between 2 and 20.

### Modelling transitions in species extinction risk

**Fig. 5:**
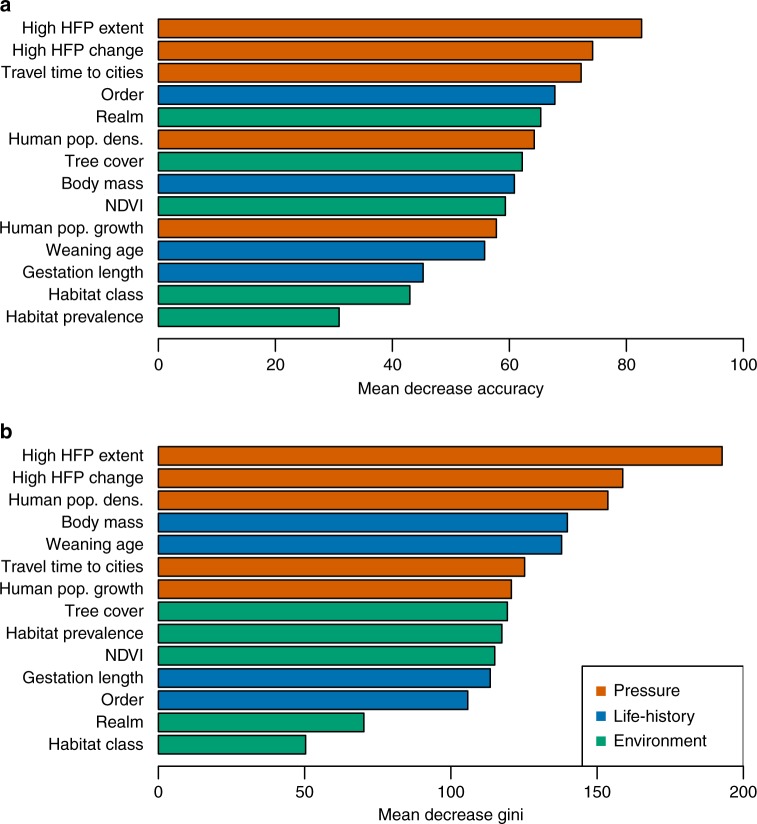
Predictive importance of variables for the prediction of extinction risk transitions in terrestrial mammals. Variables are colour-coded according to their broad class (human pressure, life-history, environmental characteristics). Different plots refer to different measures of variable importance: **a **variable effect on the overall decrease in prediction accuracy, and **b** contribution of the variable to decrease Gini Index during the classification routine. A description of all the variables can be found in Table 1. In this analysis, “high HFP" included values of 3 or above

We measured the performance of HFP in predicting low- vs. high-risk transitions in species extinction risk, using a random forest model for classification^19^. In this analysis, we compared the predictive performance of HFP with a number of other pressure, life-history, and environmental variables (Table 1). We measured HFP both as the current extent of high HFP values within species ranges, and as the change in high HFP values over the time period (1993–2009). We adopted all possible thresholds to determine low vs. high HFP values (from HFP > 0 to HFP > 49), and found that the importance of HFP variables as predictors decreased with increasing thresholds (Supplementary Fig. 6). Overall, a HFP threshold of ≥ 3 resulted in the highest prediction performance across the two HFP variables (current extent and change over time), indicating that human pressure intensification in intact and near-intact areas is globally more relevant, for explaining extinction risk transitions, than intensification within already-modified landscapes. We found that pressure variables had higher predictive performance compared to life-history and environmental variables, highlighting the magnitude of human influence on environmental trends^20^, and the two HFP variables were the most important predictors in the model (Fig. 5). The model showed good overall classification ability during cross-validation (species correctly classified = 82.9%), albeit the accuracy in predicting high-risk species (sensitivity = 60.4%) was lower than the accuracy in predicting low-risk species (specificity = 92.4%; True Skill Statistics = 0.53).

### The biogeography of human pressure within species ranges

**Fig. 6:**
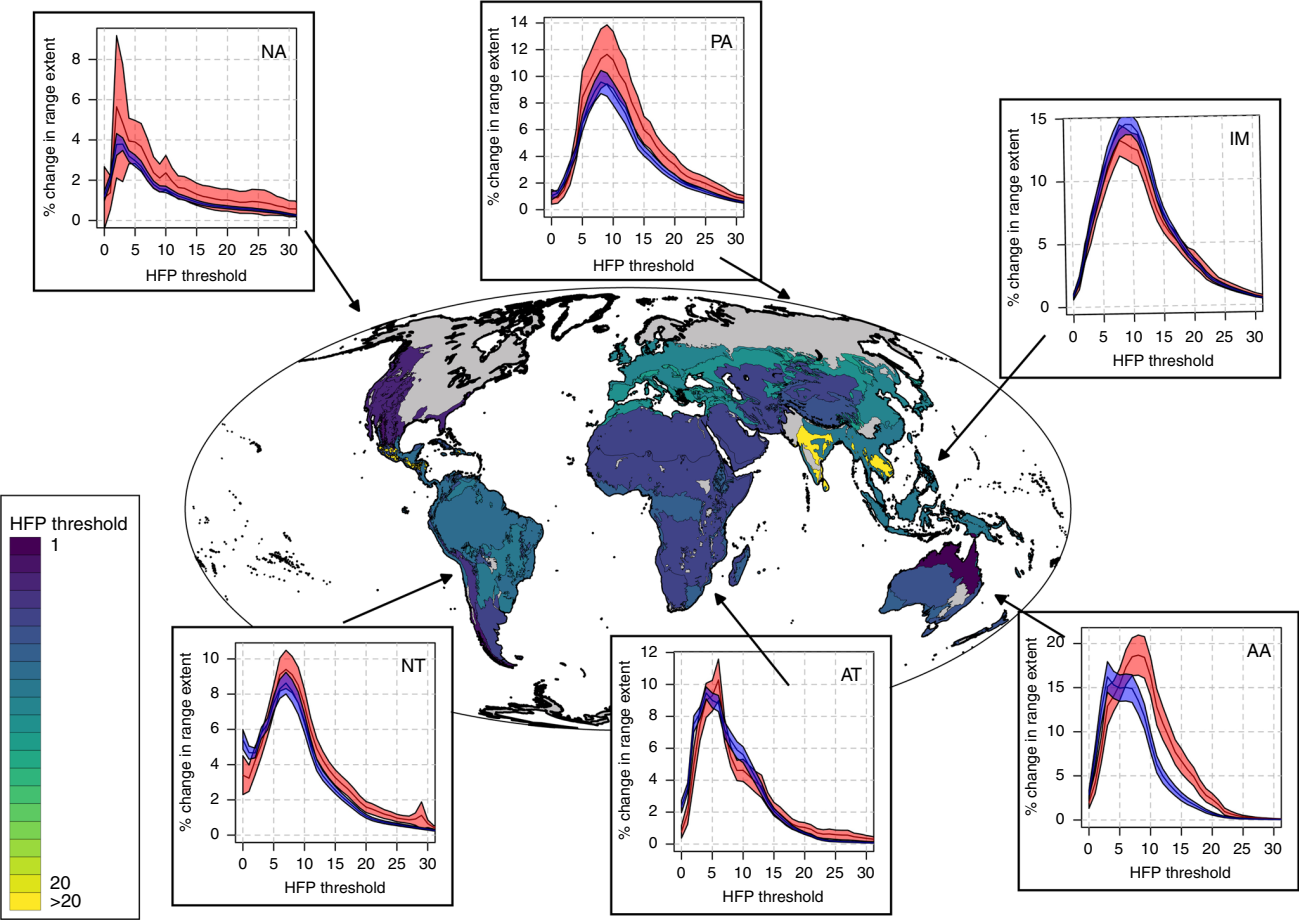
Changes in the overlap between species ranges and high human footprint (HFP) values for each biogeographic realm*. The underlying map reports, for each biome within each realm, the threshold at which change in HFP values is the highest for high-risk species. The plots report the average change in the overlap between species ranges and HFP values bigger than a given threshold within each realm (with high-risk species in red and low-risk species in blue). The shaded areas around the lines in the plots represent the 95% standard credible interval measured across a total of: 493 AA species, 854 AT species, 604 IM species, 259 NA species, 949 NT species, 442 PA species*. *Realm acronyms: AA Australasia, AT Afrotropical, IM Indomalay, NA Nearctic, NT Neotropical, PA Palearctic

We observed some differences among realms and biomes in terms of HFP change patterns (Fig. 6). For example, the HFP value at which high-risk species had the largest proportions of their ranges moving above threshold was < 6 in the Nearctic, Neotropical and Afrotropical realms, and > 6 in the Palearctic, Indomalay and Australasian realms. Most realms showed general consistency with the global analysis in that high-risk species had a higher proportion of their range moving toward higher HFP values compared to low-risk species, especially when looking at intermediate and high HFP thresholds. However there were exceptions in the Afrotropical and Indomalay realms. In the Afrotropical realm, the exception emerged for grassland biomes (Supplementary Fig. 7), where low-risk species showed larger conversions to high HFP values than high-risk species, when considering thresholds in the range 7–12. In the Indomalay realm, low-risk species had similar (or even higher) proportions of their ranges moving toward higher HFP values compared to high-risk species. This was especially the case for species living in the moist forest biome, which contrasted with the results obtained for the same biome in other realms. When looking at dry forest species in the Indomalay, we found low-risk species had faced larger increase in the extent of HFP values in the range 9–18, while high-risk species have faced a higher change for HFP values above 18.

Despite the differences in HFP change patterns observed across biogeographical domains, we still found that relatively low thresholds resulted in the highest predictive performance of “HFP change” as a variable in random forest models developed for separate realms (Supplementary Fig. 8). The lowest threshold (HFP ≥ 1) was observed in the Nearctic realm, while the highest threshold was observed in the IndoMalay realm (HFP ≥ 5).

## Discussion

In order to proactively inform monitoring and management, it is necessary to know the conditions under which a species is likely to retain an unsustainable high risk of extinction, or to face increased risk over time^8^. We found that the extent of high human pressure (as defined by the HFP index) within species ranges, and the change in this extent over time, were strong correlates of extinction risk transitions. These two variables (state and change of high HFP values) were found to be the strongest predictors of risk when compared to an array of other variables, including species’ traits, environmental conditions, and individual pressure layers. This result contrasts with the findings from previous extinction risk modelling for mammals, where the importance of human pressure as predictors was found to be lower than environmental or life-history variables^3,17,21^. This shows that temporal cumulative pressure mapping is a powerful tool for improving extinction risk modelling and forecasting, coupling changes in human pressure with changes in biodiversity state.

Identifying a threshold of human pressures beyond which species show negative response^22^ is essential for monitoring land conversion rates in the context of international biodiversity targets^15,23^. Yet the definition of “high pressure” levels has remained elusive in global analyses so far. Our results show that averting the conversion of natural and semi-natural areas, those with HFP values ≥ 3, is the most effective strategy to prevent species from undergoing a high-risk transition in their conservation status when accounting for environmental and life-history traits. These results are in line with recent findings that deforestation within intact landscapes is the strongest correlate of decline in forest species^24^, opening up the path to a number of direct threat mechanisms (such as hunting, diseases spread, and invasive species). However, protecting natural and semi-natural landscapes is not sufficient to improve the status of species which are already at a high risk, some of which have little natural habitat left within their distributions and will require habitat restoration to reduce their risk of extinction. In fact, high-risk and low-risk species showed markedly different changes in their overlap with intermediate HFP values with high-risk species facing consistently larger increase in HFP levels. This was confirmed, with an even stronger pattern, when looking at uplisted *vs* not uplisted species, albeit this latter classification could not be used for extinction risk modelling due to very high imbalance in species numbers between the two classes. The identification of this HFP threshold (i.e. 3), and what happens when changes occur in the HFP around this threshold, provide simple guidelines for identifying tipping points beyond which human activities might lead to species decline.

We found some biogeographical differences in the way low-risk and high-risk species overlap with HFP values. Particular exceptions were found in the grassland biomes of the Afrotropical realm and in the moist forest biome of the IndoMalay realm, where low-risk species faced similar (or higher) increase in HFP values compared to their high-risk counterparts. These exceptions might indicate that species living in those environments are relatively resilient to human pressure as measured in the HFP index, and respond more strongly to other pressures not incorporated in the index such as fire regimes, especially relevant in African grasslands^25^, and overexploitation, relevant in Southeast Asia^26^. However there is also the possibility that some species currently classified as low-risk might actually be facing a higher risk of extinction than previously thought, especially in forested biomes, as it seems to result from recent, rapid, deforestation^27^. These results support the call for a status re-assessment of these species within these regions. Interestingly however, when HFP change was considered as a predictor of risk within separate biogeographic realms and in combination with other variables, we found that low thresholds (in the range 1–5, depending on the realm) still performed the best in separating low from high HFP values. This demonstrates that the conservation of intact areas, and areas with little human modification, is relevant at the scale of individual realms and not only globally.

Our results showed that high-risk species had faced larger increases in pressure levels within areas of moderate HFP values, while low-risk species had faced larger pressure increases in areas of former low HFP (those < 2). This includes the loss of wilderness areas, which was more likely to occur within the range of low-risk species than high-risk species. This finding is probably related to the fact threatened species are less likely to overlap with wilderness areas compared to non-threatened species, as a reflection of pressure operating within their past ranges. This finding means that the continuous conversion of intact and near-intact areas will likely result in species that are currently classified as low-risk to become high-risk in the future. The loss of intact lands within the ranges of low-risk species should thus act as an early indication of a trajectory of increasing species endangerment, and points to the need of identifying, and securing, those remaining intact ecosystems.

Our model was better able to correctly classify low-risk species than high-risk species. This might be related the fact that some high-risk species are responding to different components of pressures, not well represented here, while low-risk species are just not facing significant pressures. However this same pattern was also found (at various degrees) in other extinction risk modelling exercises^3,7,8,28^, indicating that the condition under which species are likely to undergo a low-risk transition are likely easier to identify compared to the conditions leading to high-risk transitions. When human pressure is operating, the risk for species is determined by a complex combination of pressure levels, species’ sensitivity and their potential for adaption^29^, which determine higher levels of uncertainty in the predictions. This adds a level of complexity in understanding the relationship between pressure change and change in species extinction risk.

The current availability of HFP maps (for the years 1993 and 2009) allowed us to test the relationship between human pressure and extinction risk over a similar time frame, that encompasses three or more generations for 73% species in our analyses^30^. This relationship between change in HFP and change in extinction risk can serve as a basis for future research. For example, our modelling framework, calibrated on observed trends, can allow projecting future extinction risk transitions under alternative scenarios of socio-economic development^31^. This will require generating future projections of the base pressure layers that constitute the HFP map, as well as predicting the shift in species distributions due to future climate and land-use change^32^. Also, the established relationship between HFP and extinction risk transitions can be used to estimate the risk faced by ‘data deficient’ species^28,33^, but this would require resolving the taxonomic and geographical uncertainty characterising species in this category.

Efforts to integrate human pressure maps and extinction risk has the potential to change the way we assess species risk^9^ and proactively inform conservation action in a way that minimises the number of species that will face a high risk of decline. Conservation organisations that have a mission to prevent the decline of species can use our approach to prioritise actions for minimizing extinction risk. These include both species that are already threatened with extinction and species that are likely to become so if current rates of intensification in the human footprint continue into the future. The HFP index presents a standardised representation of human pressure levels, combining different human activities that represent potential sources of impact for species. While this index represents a comprehensive and easy tool for estimating change in species extinction risk and guide broad-scale conservation efforts, we acknowledge that it cannot substitute local-scale assessments of the conservation needs of each species. Instead, knowing which species and which areas are most likely to face a high risk can guide the prioritisation of local-scale assessments by conservation practitioners.

## Methods

### Human footprint state and change

We used the recent release of the global HFP map^14,16^, to represents the cumulative human impact on the environment. This map is built from eight base layers: (i) the extent of built environments; (ii) crop land; (iii) pasture land; (iv) human population density; (v) night-time lights; (vi) railways; (vii) roads; and (viii) navigable waterways. Following the approach originally proposed by Sanderson and colleagues^34^, each layer was placed in a 1–10 scale with a value weighted according to the relative intensity of human pressure (see Venter et al.^14^ for full justification and validation): (i) all built environments were assigned a score of 10 while non-built environment had a score of zero); (ii) areas mapped as croplands were assigned a score of 7; (iii) areas mapped as pasture lands were assigned a score of 4; (iv) areas with a high human population density of > 1,000 people/km^2^ received a score of 10, while areas with lower density received a lower log-scaled score; (v) areas were divided into 10 quantiles of increased night-time light intensity associated to score of 1 to 10, while areas with no lights were assigned a zero; (vi) railways and their immediate 500 m buffers were given a score of 8, with a value of zero elsewhere (i.e. assuming no indirect impact); (vii) roads and their immediate 500 m buffers were given a score of 8 (direct impact), while nearby areas up to 15 km had score that decayed exponential to zero (indirect impact); (viii) areas adjacent to navigable water bodies were assigned a score of 4, which decayed exponentially out to 15 km away from the waters. After each pressure layer was standardised within the same values range, they were summed together to create a cumulative map of human pressure. The results are two globally standardised HFP maps, with values ranging from 0 to 50 and a spatial resolution of 1 km^2^, one for the year 1993 and one for 2009 (based on pressure layers referred to the different periods). In this analysis we use the integer version of the HFP maps, to only represent integer changes in the index (+ 1, + 2, + 3 etc.).

We measured the change in HFP values for each 1 km^2^ terrestrial grid cell between 1993 and 2009, and contrasted this change with species geographic distributions to understand changes in perceived treat levels for the species. HFP change can result in an increase in pressure level, i.e. from lower to higher values, or a decrease in pressure, i.e. the opposite. Here we only accounted for increases in HFP values, as decreases in pressure levels (e.g. abandonment of agricultural land) are likely to take time before having a measurable effect on species threat status, especially for species with a long generation time such as some of the high-risk species in our dataset^30^ (average generation time for high-risk species is 7 years, compared to 4.5 years for low-risk species). We used HFP and its change over time as a predictor of extinction risk change, in combination with previously identified variables (Table 1).

### Species extinction risk change

We represented the extinction risk of terrestrial mammal species using the information available from the IUCN Red List^35^, and the retrospective Red List Assessments published in Hoffmann et al.^13^. We considered the 2010 IUCN Red List categories of each species and the retrospectively assigned categories for 1996. These latter categories were defined using the same methodology as the 2010 assessments, but referred to the past condition of the species. We considered the following IUCN Red List categories, assigned using a set of five quantitative criteria with associated sub-criteria and thresholds^36^: Least Concern (LC); Near Threatened (NT); Vulnerable (VU); Endangered (EN); Critically Endangered (CR); Extinct in the Wild (EW); Extinct (EX). We excluded species not evaluated in the Red List, those without a defined risk of extinction category (Data Deficient), and those already extinct at the beginning of the study period. We retained 4421 species of terrestrial mammals with a defined extinction risk category for the years 1996 and 2010, corresponding to 83% of all species in the group.

We recorded the initial (1996) and final (2010) Red List category of each species, and followed Di Marco et al.^8^ in classifying species into two main groups (Fig. 1): low-risk transitions and high-risk transitions. The low-risk group included species that were LC throughout the study period, together with species that moved from any higher Red List category to a lower category (i.e. category ‘downlisting’). The high-risk group included all species that were originally threatened or near threatened and retained their category throughout the study period, together with species that moved from any lower Red List category to a higher category (i.e. category ‘uplisting’). The method behind this classification has been statistically justified^8^, and reflects the fact that remaining within the same Red List category through time does not necessarily imply that a species is in a stable condition. For instance, while a species that retains a LC category is not undergoing a significant population decline (or loss of geographic range), a species retaining a threatened category implies substantial continued decline^36^.

We also tested the use of a more conservative approach for classifying extinction risk transitions, where species were separated into two groups: ‘uplisted’ species, those that had a deterioration in their Red List category (eg from Least Concern to Near Threatened), and ‘not uplisted’ species, those that retained the same category or improved it. This classification can be seen as a more conservative approach for defining extinction risk transitions, because in this case the ‘high-risk’ group only includes transitions that are of sufficient magnitude to generate an upward shift in Red List categories. This classification however generated a large imbalance between species groups, with only ~4% of species being included in the uplisted class.

### Human footprint as a driver of extinction risk change

Several methods are available to measure the level of overlap between a spatial pressure layer and a species’ geographic range^22^. These include both measures of central tendencies, e.g. the mean/median pressure level observed within the range, and measures of spatial extent, e.g. how much of the species range is covered with high pressure levels. Measuring the extent of high pressure levels within a species’ range has been shown to be a more sensitive way to predict extinction risk than using mean pressure levels^22^ and was often a preferred choice in comparative extinction risk modelling^21,37^. However, identifying the best way for separating low and high pressure levels requires testing multiple thresholds.

We measured the cumulative overlap between 1993 and 2009 HFP values within species ranges, generating curves to represent how much of a species’ range overlaps with increasing values of HFP (from 0 to 50). We used the same species distribution range maps for these measures, since past range maps for the ~4500 species included in our analyses were not available. Given our study period was reasonably restricted (16 years), we assumed change in the extent of species geographic range was overall limited. We generated separate curves to represent the average accumulation of HFP values in low-risk and high risk species, both in 1993 and 2009. We then tested all possible thresholds of HFP (from HFP > 0 to HFP > 49) to define the change in the overlap between species ranges and high HFP values between 1993 and 2009. We measured the proportional difference, between 1993 and 2009, in the overlap of species ranges with HFP values smaller or equal than the defined threshold. We also measured the changes in the extension of high HFP values after discarding areas where HFP values were lower in 2009 than in 1993 (assuming ‘no change’ in those cases). We reported the effect size of the extent of high HFP values in low-risk vs. high-risk species, and the effect size of the change in the extent of high HFP values in low-risk vs. high-risk species, using *Cohen’s d* statistic^38^.

We used HFP change to predict species extinction risk transitions (low-risk vs high-risk), using a multi-variable Random Forest model^3,7,8^. Following previous works^8,39^, we included important intrinsic and extrinsic predictors of risk (see Table 1 for a description). In identifying extrinsic variables, we favoured those datasets with a good temporal match with the period over which extinction risk transitions were observed. We did not include species’ range size as a predictor, in order to prevent potential circularity in the estimation of extinction risk^4^. We used as predictors both the current extent of high HFP values within species’ ranges, and the proportional change in the extent of high HFP values through time. For example, we measured how much of the distribution range of the lion (*Panthera leo*) is currently in overlap with HFP values of *x* or above, and which proportion of the lion’s range has undergone a change from low ( < *x*) to high ( ≥ *x*) values of HFP during 1993–2009. We measured the importance of HFP as a predictor relative to other variables, using two standard metrics for Random Forest models^19^. The first metric is the decrease in classification accuracy, reporting the decrease in the model’s ability to correctly classify data if the values of a predictor variable are randomly permuted. The second metric is the decrease in Gini coefficient, reporting the total decrease in Gini coefficient from splitting the data based on a predictor variable, averaged over all classification trees in the Random Forest. We also report the overall performance of our Random Forest model during cross-validation, in terms of: proportion of correctly classified species, proportion of correctly classified high-risk species (sensitivity), proportion of correctly classified low-risk species (specificity), and true skill statistic^40^ (TSS = sensitivity + specificity −1).

We tested the use of a Random Forest model to predict uplisted *vs* not uplisted species, but found completely biased results with almost all species being classified as not uplisted. This implies the model is unable to correctly classify uplisted species, due to the very large imbalance between number of uplisted species (4% of the total) and number of not uplisted species (96% of the total). We thus only report the main results on the low-risk *vs* high-risk model.

### Measuring human footprint impact across biogeographic realms

We represented the biogeographical variation in HFP change, by measuring the change in overlap of species ranges with high HFP values (again testing all HFP thresholds) within separate biogeographical domains. We run separate analyses for separate biogeographic realms, and for separate biomes within each realm (i.e. biome-realms), following the biogeographic classification of the world proposed by Olson et al.^41^. In this case we only retained species with > 50% of their distributions within a realm, or a biome-realm, and we discarded all biome-realms which did not have at least five low-risk and five high-risk species. We also run separate Random Forest models for species restricted to separate biogeographic realms, following the same settings as in the global model (see previous section).

All spatial analyses were performed in GRASS GIS^42^, statistical analyses were performed in R^43^, using the packages ‘effsize’^44^ and ‘randomForests’^45^.

## Electronic supplementary material

Supplementary Information

## Data Availability

The Human Footprint dataset used in this study is available from the Dryad Digital Repository with the identifier doi:10.5061/dryad.052q5^14^. The other datasets that support the findings of this study derive from published sources, cited in the Methods section and listed in Table 1.

## References

[1] De Vos JM, Joppa LN, Gittleman JL, Stephens PR, Pimm SL (2015). Estimating the normal background rate of species extinction. Conserv. Biol..

[2] Brook BW, Sodhi NS, Bradshaw CJA (2008). Synergies among extinction drivers under global change. Trends Ecol. Evol..

[3] Murray KA, Verde Arregoitia LD, Davidson A, Di Marco M, Di Fonzo MMI (2014). Threat to the point: improving the value of comparative extinction risk analysis for conservation action. Glob. Chang. Biol..

[4] Purvis A, Gittleman JL, Cowlishaw G, Mace GM (2000). Predicting extinction risk in declining species. Proc. R. Soc. Lond. B.

[5] Fisher DO, Blomberg SP, Owens IPF (2003). Extrinsic versus intrinsic factors in the decline and extinction of Australian marsupials. Proc. R. Soc. B.

[6] Cardillo M (2005). Multiple causes of high extinction risk in large mammal species. Science.

[7] Davidson AD, Hamilton MJ, Boyer AG, Brown JH, Ceballos G (2009). Multiple ecological pathways to extinction in mammals. Proc. Natl. Acad. Sci. U. S. A..

[8] Di Marco M, Collen B, Rondinini C, Mace G (2015). Historical drivers of extinction risk: using past evidence to direct future monitoring. Proc. R. Soc. B.

[9] Rondinini C, Di Marco M, Visconti P, Butchart S, Boitani L (2014). Update or outdate: long term viability of the IUCN Red List. Conserv. Lett..

[10] Bland LM (2015). Cost-effective assessment of extinction risk with limited information. J. Appl. Ecol..

[11] Cardillo M, Meijaard E (2012). Are comparative studies of extinction risk useful for conservation?. Trends Ecol. Evol..

[12] Di Marco M (2014). A Retrospective evaluation of the global decline of carnivores and ungulates. Conserv. Biol..

[13] Hoffmann M (2010). The impact of conservation on the status of the world’s vertebrates. Science.

[14] Venter O (2016). Global terrestrial human footprint maps for 1993 and 2009. Sci. Data.

[15] Watson James E.M., Shanahan Danielle F., Di Marco Moreno, Allan James, Laurance William F., Sanderson Eric W., Mackey Brendan, Venter Oscar (2016). Catastrophic Declines in Wilderness Areas Undermine Global Environment Targets. Current Biology.

[16] Venter O (2016). Sixteen years of change in the global terrestrial human footprint and implications for biodiversity conservation. Nat. Commun..

[17] Verde Arregoitia LDBiases (2016). gaps, and opportunities in mammalian extinction risk research. Mamm. Rev..

[18] Watson JEM (2016). Persistent disparities between recent rates of habitat conversion and protection and implications for FUture Global Conservation Targets. Conserv. Lett..

[19] Breiman L (2001). Random Forests.

[20] Santini L, González-Suárez M, Rondinini C, Di Marco M (2017). Shifting baseline in macroecology? Unravelling the influence of human impact on mammalian body mass. Divers. Distrib..

[21] Cardillo M (2008). The predictability of extinction: biological and external correlates of decline in mammals. Proc. R. Soc. B.

[22] Di Marco, M., Rondinini, C., Boitani, L. & Murray, K. A. Comparing multiple species distribution proxies and different quantifications of the human footprint map, implications for conservation. Biol. Conserv. 165, 203–211 (2013).

[23] Jones KR (2018). One-third of global protected land is under intense human pressure. Science.

[24] Betts MG (2017). Global forest loss disproportionately erodes biodiversity in intact landscapes. Nature.

[25] Giglio L, Randerson JT, Van Der Werf GR (2013). Analysis of daily, monthly, and annual burned area using the fourth-generation global fire emissions database (GFED4). J. Geophys. Res. Biogeosciences.

[26] Sodhi NS, Koh LP, Brook BW, Ng PKL (2004). Southeast Asian biodiversity: an impending disaster. Trends Ecol. Evol..

[27] Tracewski Ł (2016). Toward quantification of the impact of 21st-century deforestation on the extinction risk of terrestrial vertebrates. Conserv. Biol..

[28] Bland LM, Collen B, Orme CDL, Bielby J (2015). Predicting the conservation status of data-deficient species. Conserv. Biol..

[29] Di Marco M (2012). A novel approach for global mammal extinction risk reduction. Conserv. Lett..

[30] Pacifici M (2013). Generation length for mammals. Nat. Conserv.

[31] Riahi K (2017). The shared socioeconomic pathways and their energy, land use, and greenhouse gas emissions implications: an overview. Glob. Environ. Chang.

[32] Visconti P (2016). Projecting global biodiversity indicators under future development scenarios. Conserv. Lett..

[33] Jetz W, Freckleton RP (2015). Towards a general framework for predicting threat status of data-deficient species from phylogenetic, spatial and environmental information. Philos. Trans. R. Soc. B.

[34] Sanderson EW (2002). The human footprint and the last of the wild. Bioscience.

[35] IUCN. IUCN Red List of threatened species version 2012.1. Version 20104 2008, (2012).

[36] IUCN. IUCN Red list categories and criteria, version 3.1. (IUCN Gland, Switzerland and Cambridge, UK, 2001).

[37] Di Marco M (2014). Drivers of extinction risk in African mammals: the interplay of distribution state, human pressure, conservation response and species biology. Philos. Trans. R. Soc. Lond. B Biol. Sci..

[38] Cohen, J. Statistical Power Analysis for the Behavioral Sciences (2nd ed.). (Academic Press, New York, 1988).

[39] Di Marco M, Santini L (2015). Human pressures predict species’ geographic range size better than biological traits. Glob. Chang. Biol..

[40] Allouche O, Tsoar A, Kadmon R (2006). Assessing the accuracy of species distribution models: prevalence, kappa and the true skill statistic (TSS). J. Appl. Ecol..

[41] Olson DM (2001). Terrestrial ecoregions of the world: a new map of life on earth. Bioscience.

[42] GRASS Development Team. Geographic Resources Analysis Support System (GRASS) Software, Version 7.0. (2016).

[43] R. Core Team. R: A language and environment for statistical computing. R Foundation for Statistical Computing. (2015).

[44] Torchiano, M. effsize: Efficient Effect Size Computation. R package version 0.7.1. (2017). Available at: https://cran.r-project.org/package=effsize.

[45] Liaw A, Wiener M (2002). The randomforest package. R. News.

[46] CIESIN & CIAT. Gridded Population of the World, Version 3 (GPWv3): Population Density Grid. Palisades, NY: NASA Socioeconomic Data and Applications Center (SEDAC) (2005). Available at: http://sedac.ciesin.columbia.edu/data/set/gpw-v3-population-density. (Accessed: 1st November 2013)

[47] CIESIN, FAO & CIAT. Gridded Population of the World, Version 3 (GPWv3): Population Count Grid, Future Estimates. Palisades, NY: NASA Socioeconomic Data and Applications Center (SEDAC) (2005). Available at: http://sedac.ciesin.columbia.edu/data/set/gpw-v3-population-count-future-estimates. (Accessed: 1st November 2013)

[48] Nelson, A. Travel time to major cities: a global map of Accessibility. Global Environment Monitoring Unit - Joint Research Centre of the European Commission, Ispra Italy. (2008). Available at: http://bioval.jrc.ec.europa.eu/products/gam/index.htm.

[49] Jones KE (2009). PanTHERIA: a species-level database of life history, ecology, and geography of extant and recently extinct mammals. Ecology.

[50] Tacutu R, Craig T, Budovsky A (2013). Human ageing genomic resources: integrated databases and tools for the biology and genetics of ageing. Nucleic Acid. Res.

[51] Verde Arregoitia L, Blomberg S, Fisher D (2013). Phylogenetic correlates of extinction risk in mammals: species in older lineages are not at greater risk. Proc. R. Soc. B.

[52] Wilman H (2014). EltonTraits 1. 0: species-level foraging attributes of the world’s birds and mammals. Ecology.

[53] Rondinini C (2011). Global habitat suitability models of terrestrial mammals. Philos. Trans. R. Soc. Lond. B. Biol. Sci..

[54] NASA Earth Observatory Group (2018) Normalized difference vegetation index. Retrieved from http://neo.sci.gsfc.nasa.gov/view.php?datasetId=MOD_NDVI_M.

[55] Hansen MC (2013). High-resolution global maps of 21st-century forest cover change. Science.

