# Supplementary material for: Changes in human footprint drive changes in species extinction risk
Source: Nat Commun. 2018 Nov 5;9:4621. doi: 10.1038/s41467-018-07049-5 (PMC6218474; doi:10.1038/s41467-018-07049-5)
Supplement: Supplementary file 1 — Supplementary Information [file 41467_2018_7049_MOESM1_ESM.pdf]

**SUPPLEMENTARY INFORMATION FOR**

**Di Marco et al.**

**Change in human footprint drives change in species extinction risk**

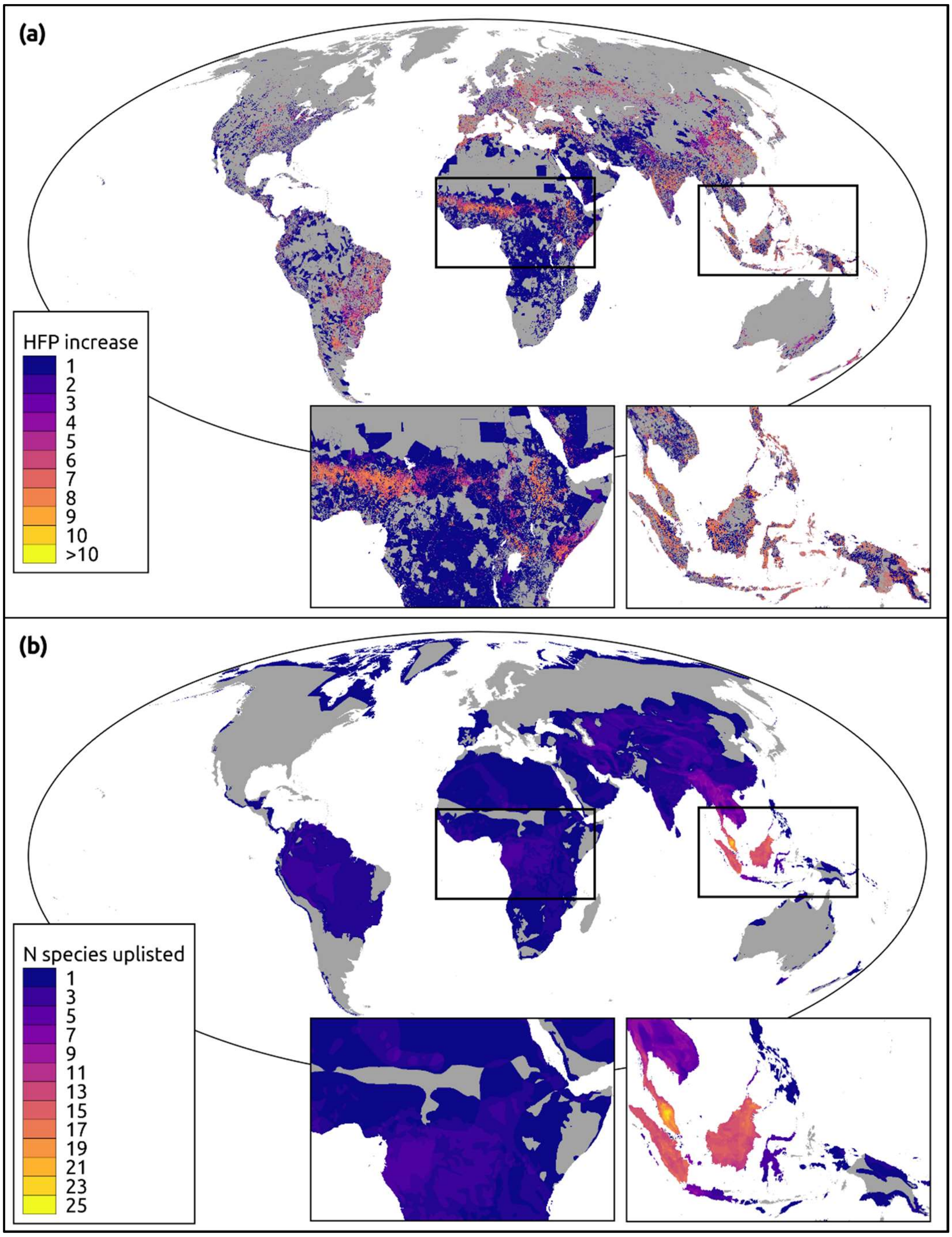


**Supplementary Fig. 1** Maps showing the location of areas that faced an increase in human footprint (HFP) values during the study period (panel a), and areas where one or more species have been 'uplisted' from any Red List category to a higher Red List category (panel b).

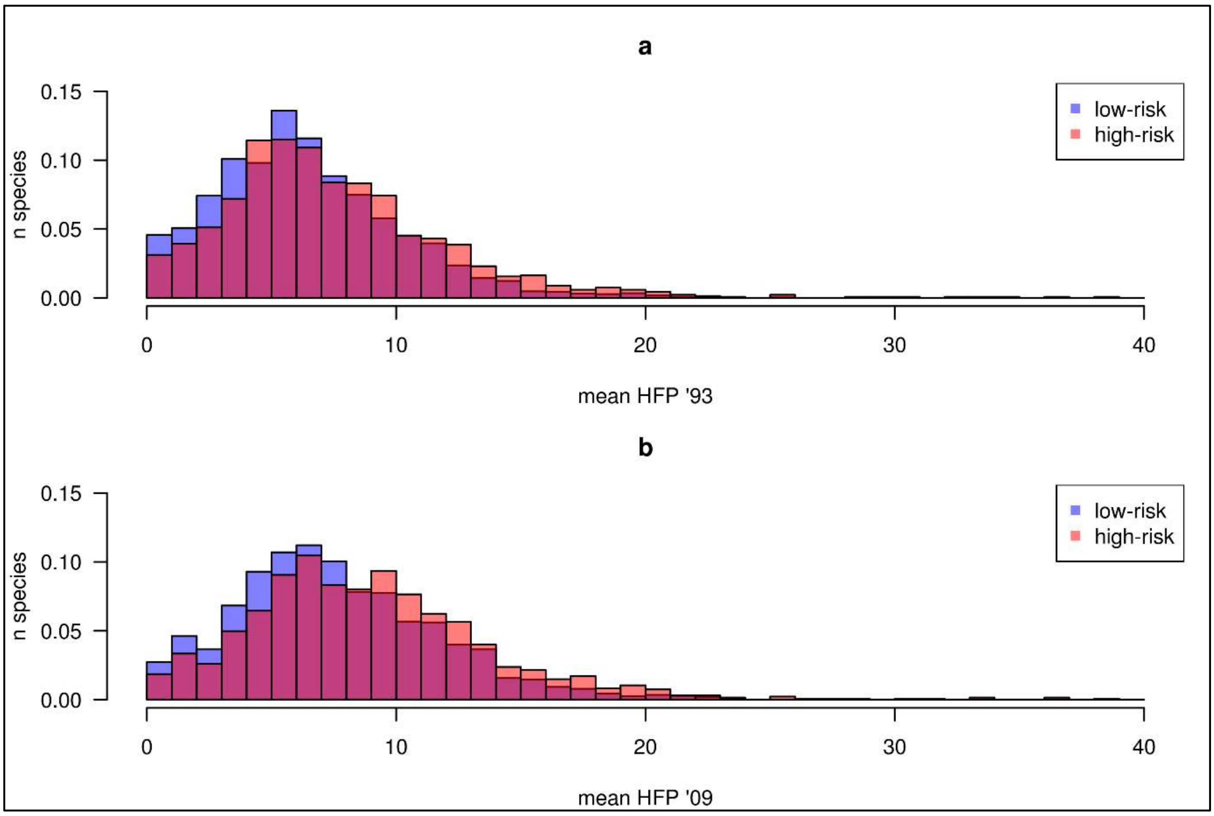


**Supplementary Fig. 2** Frequency distribution of mean Human Footprint (HFP) associated with high-risk species and low-risk species in 1993 (a) and 2009 (b).

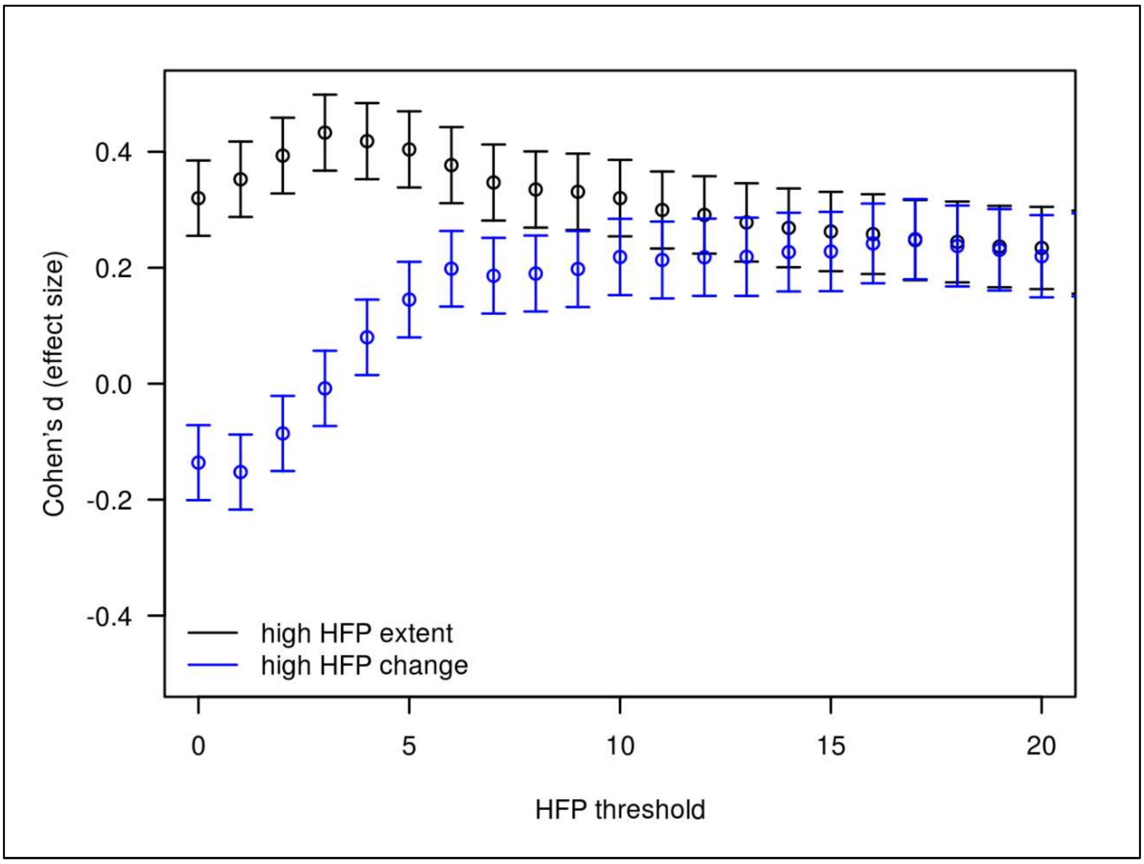


**Supplementary Fig. 3** Effect size (Cohen's d) of the difference in the extent of high human footprint values and the change in such extent over time, between low-risk and high-risk species. Effect size values (y axis) are reported for "high human footprint" thresholds in the range >0 to >20 (x axis). The error bars represent the 95% standard credible interval measured across a total of 4,421 species.

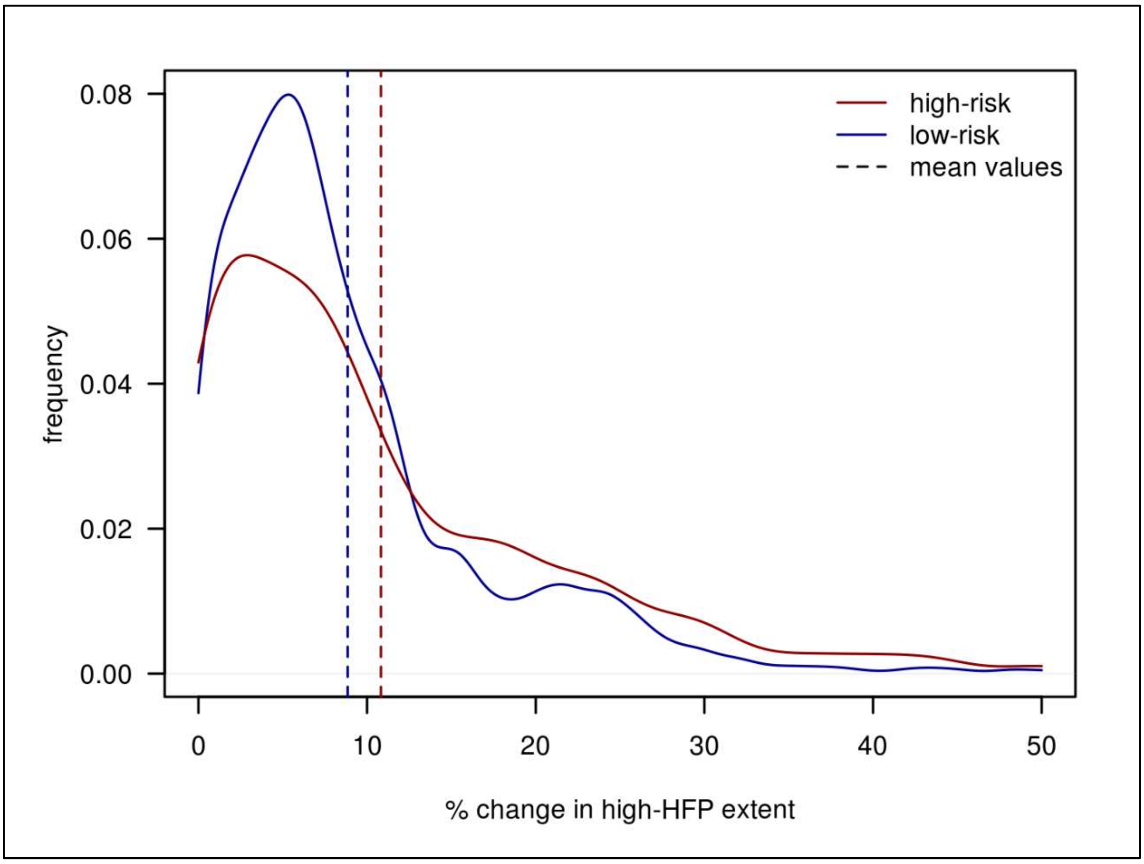


**Supplementary Fig. 4** Frequency distribution of percentage change in the extent of high human footprint values within the geographic range of species, with high-risk species in red and low-risk species in blue. In this analysis, "high HFP values" were classified as 6 or above.

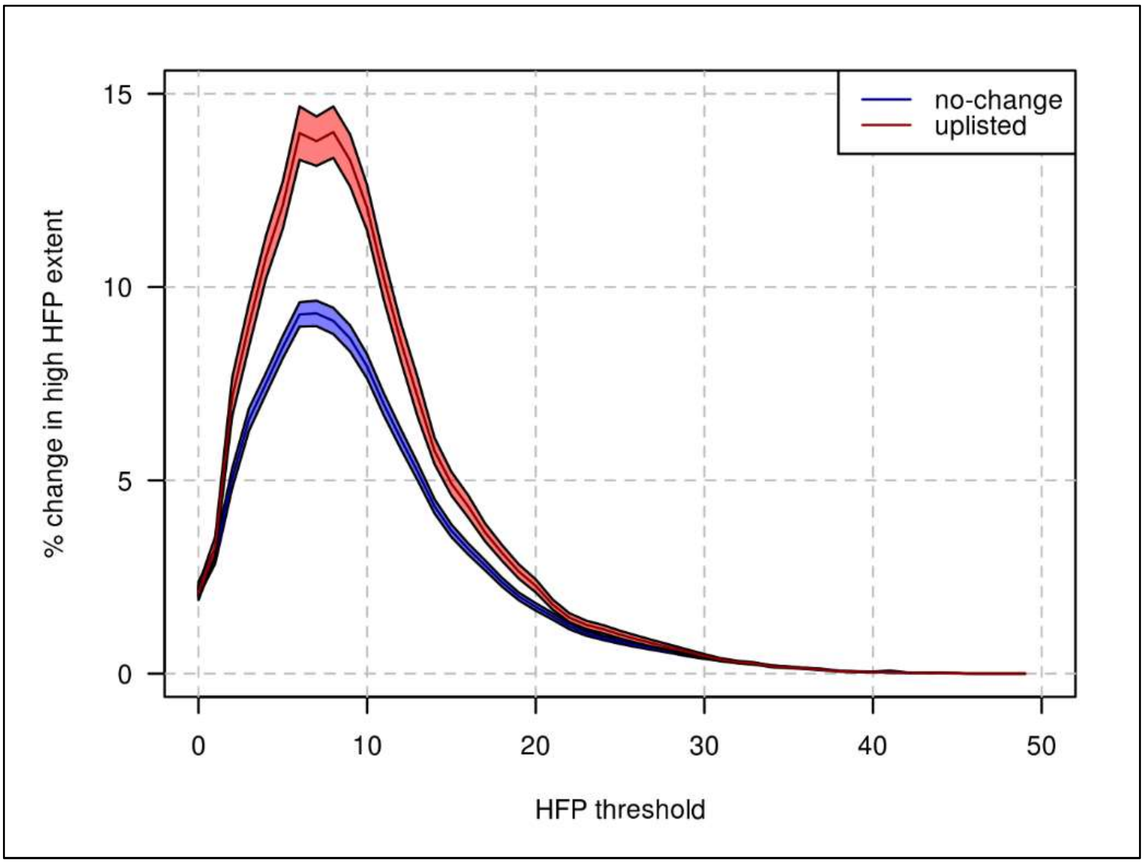


**Supplementary Fig. 5** Changes in the overlap between species ranges and high human footprint values over time. The lines report the average change in the overlap between species ranges and human footprint values bigger than any given threshold. The red line refers to species that faced an uplisting of their Red List category during the study period (red), the blue line refers to species that did not face an uplisting in their category. The shaded areas around the lines represent the 95% standard credible interval measured across a total of 4,421 species.

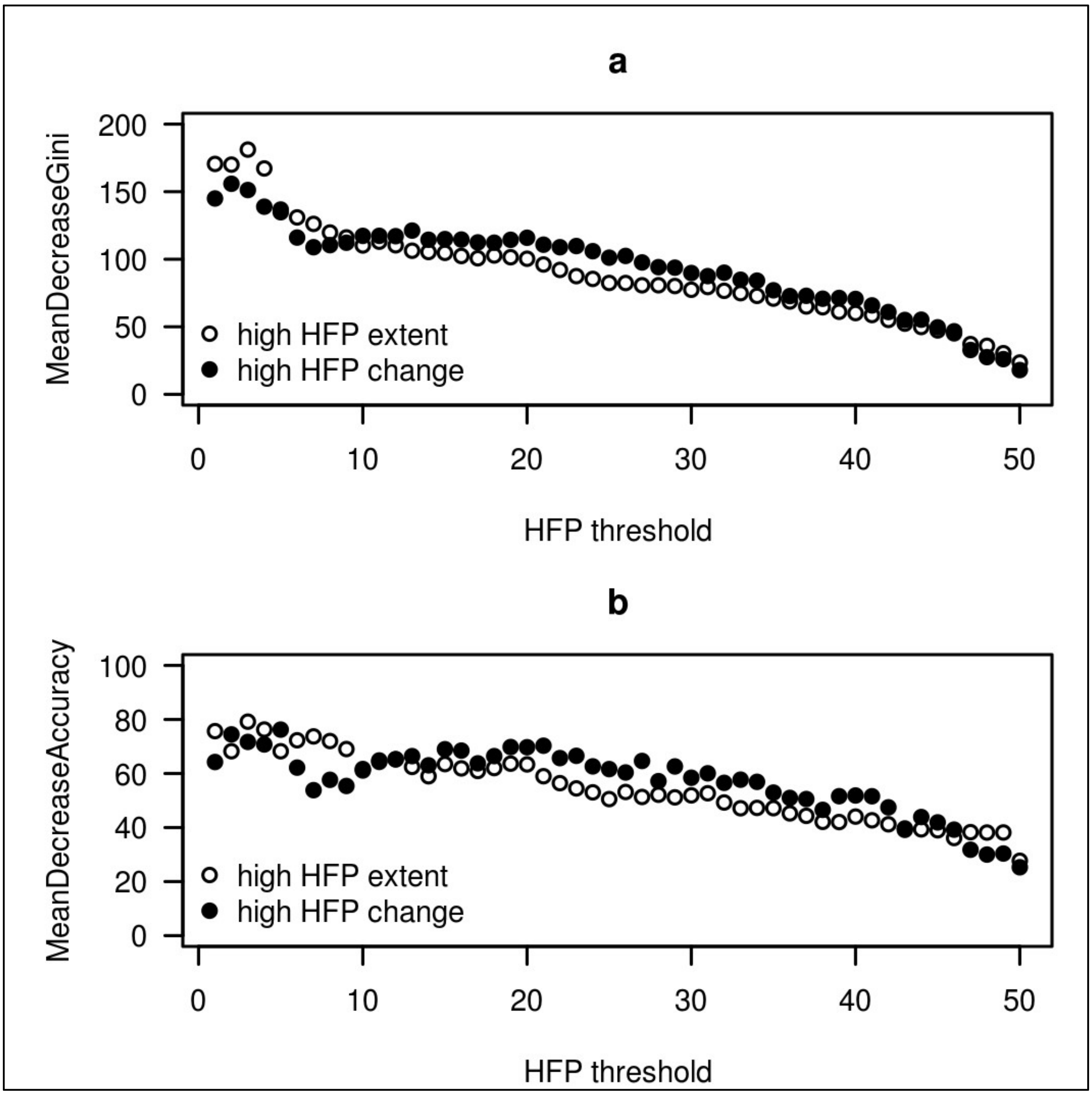


**Supplementary Fig. 6** Predictive importance of human footprint in a Random Forest model for the prediction of extinction risk transitions, when different thresholds are adopted to define the extent of high HFP values within species ranges. For each HFP threshold, the current extent of high HFP values within species ranges and the proportional difference in the extent of high HFP value between 1993 and 2009 are measured, after removal of HFP improvements. Panels a) and b) refer to different metrics of importance, as detailed in methods.

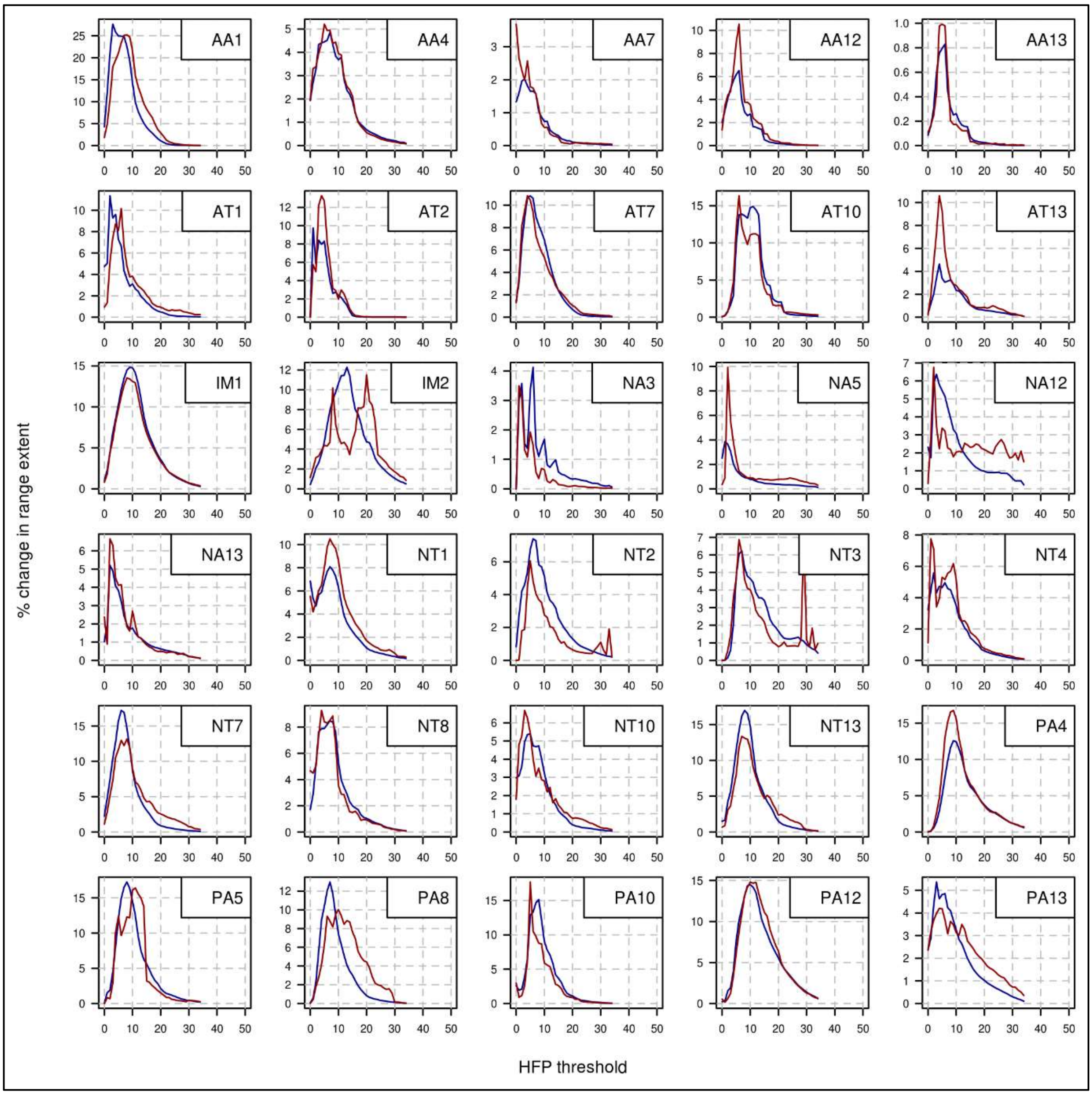


**Supplementary Fig. 7** Changes in the overlap between species ranges and high Human Footprint (HFP) values over time, for each combination of biome* and realm† with at least 10 low-risk and 10 high-risk species.

*Biome numbers: 1) Tropical & Subtropical Moist Broadleaf Forests, 2) Tropical & Subtropical Dry Broadleaf Forests, 3) Tropical & Subtropical Coniferous Forests, 4) Temperate Broadleaf & Mixed Forests, 5) Temperate Conifer Forests, 6) Boreal Forests/Taiga, 7) Tropical & Subtropical Grasslands, Savannas & Shrublands, 8) Temperate Grasslands, Savannas & Shrublands, 9) Flooded Grasslands & Savannas, 10) Montane Grasslands & Shrublands, 11) Tundra, 12) Mediterranean Forests, Woodlands & Scrub, 13) Deserts & Xeric Shrublands, 14) Mangroves.

†Realm acronyms: NA Nearctic, PA Palearctic, IM Indomalay, AA Australasia, AT Afrotropical, NT Neotropical.

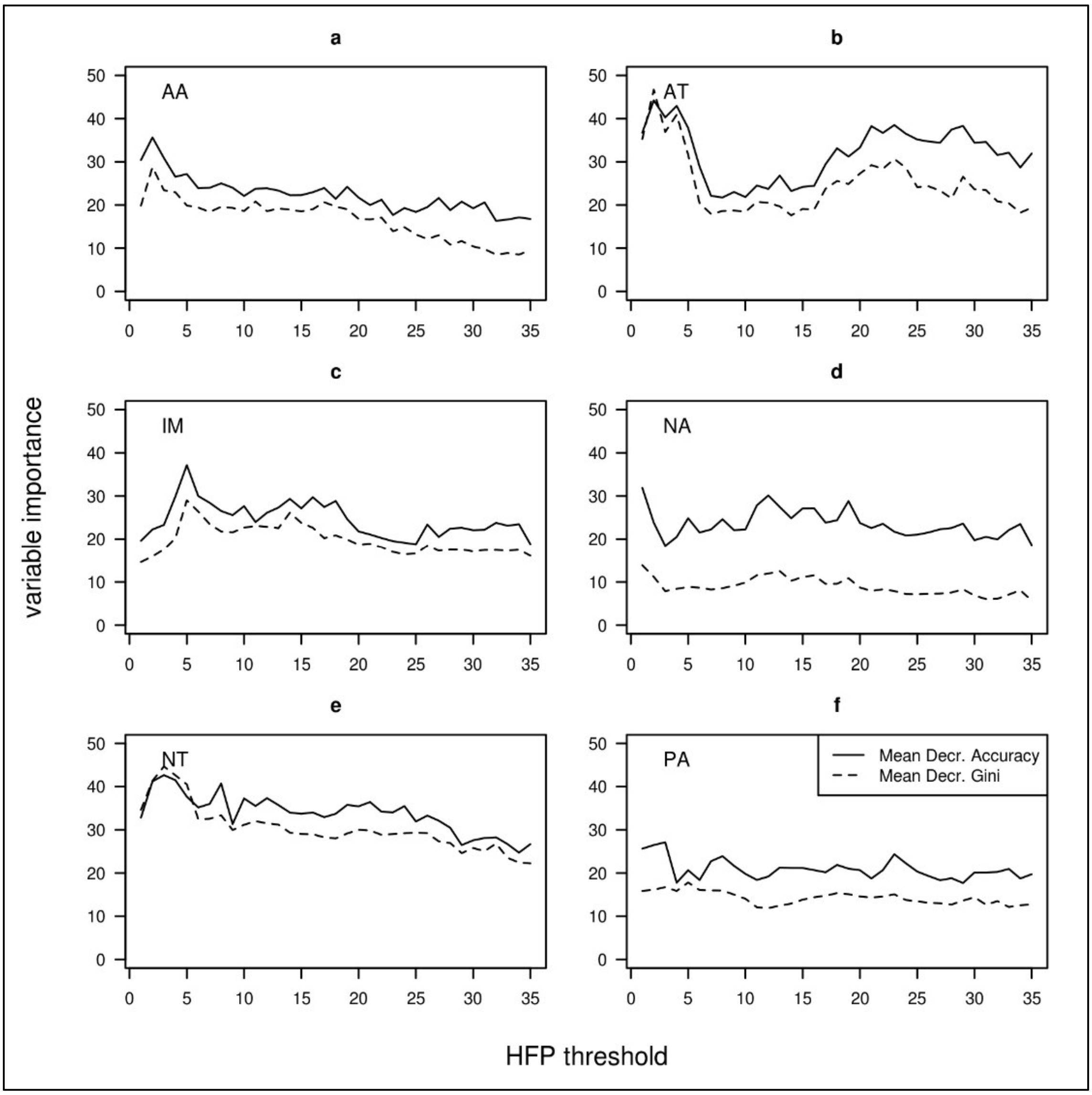


**Supplementary Fig. 8** Predictive importance of human footprint in a Random Forest model for the prediction of extinction risk transitions, when different thresholds are adopted to define the extent of high HFP values within species ranges. For each HFP threshold, the proportional difference in the extent of high HFP value between 1993 and 2009 are measured, after removal of HFP improvements. Panels (a) to (f) refer to models run for species restricted to different biogeographic realms*. Two measures of variable importance are reported, as detailed in methods.

*Realm acronyms: NA Nearctic, PA Palearctic, IM Indomalay, AA Australasia, AT Afrotropical, NT Neotropical.
